# Left atrial and left atrial appendage 4D blood flow dynamics in atrial fibrillation

**DOI:** 10.1186/1532-429X-18-S1-O90

**Published:** 2016-01-27

**Authors:** Michael Markl, Charles Foucar, Maria L Carr, Daniel C Lee, Jason Ng, James C Carr, Jeffrey J Goldberger

**Affiliations:** grid.465264.7Northwestern University, Chicago, IL USA

## Background

Atrial fibrillation (AF) is associated with increased risk of stroke due to development of left atrial appendage (LAA) thrombus. Clinical risk scores (CHA_2_DS_2_-VASc) used to assess thromboembolic risk have limited predictive value. Studies have shown that physiologic factors such as decreased LAA velocity and increased stasis are associated with thrombus formation in the LAA and may be better predictors for stroke. Currently available diagnostic tools such as TEE, however, are limited as they do not completely assess the complex 3D LAA blood flow and are invasive. As a result, the impact of AF on global and regional atrial 3D flow dynamics is poorly understood. In addition, it is unclear if the stimulus for LAA thrombus formation is driven by hemodynamic changes in the LAA alone or more broadly associated with AF induced flow alterations affecting the entire left atrium (LA). It was the aim of this study to test the potential of novel 4D flow MRI tools (anatomic maps of LA stasis, peak velocity, and time-to-peak velocity) for the characterization of LA and LAA flow dynamics.

## Methods

**Figure 1:**
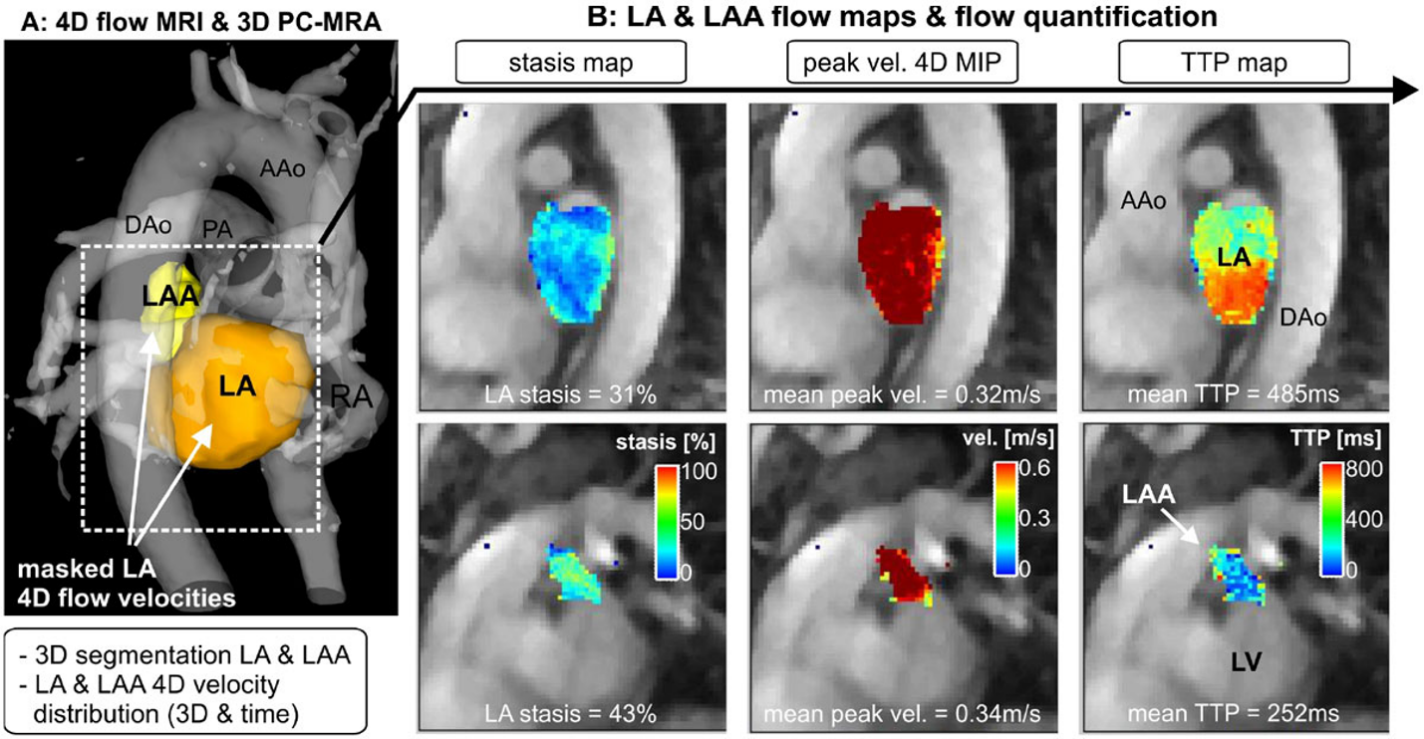
**4D flow MRI (A) and derived 3D PC angiogram (PC-MRA) in a male AF patient (EF = 57%, heart rate = 46 bpm, CHA**_**2**_**DS**_**2**_**-VASc score = 1, in sinus rhythm during the MRI scan)**. 3D segmentation of the LA and LAA was used to mask the measured time-resolved 3-directional blood flow velocities inside the LA and LAA boundaries for the calculation of LA and LAA stasis, peak velocity and time-to-peak (TTP) maps. (B)AAo/Dao: ascending/descending aorta, RA: right atrium, LV: left ventricle, PA: pulmonary artery.

4D flow MRI was employed to measure in-vivo time-resolved 3D blood flow velocities in 75 subjects: 30 AF patients in sinus rhythm (AF-sinus), 30 AF patients in AF (AF-afib) and 15 normal controls. Data analysis included 3D segmentation of the LA and LAA to isolate the velocity data in the LA and LAA (figure 1). Absolute atrial velocities were calculated for each voxel and time frame inside the LA and LAA and used to derive LA and LAA stasis, peak velocity and time-to-peak (TTP) maps (figure 1B). Peak velocity (5% of max velocity), mean velocity, and stasis (number of voxels with vel. < 0.1 m/s) were quantified.

## Results

**Figure 2:**
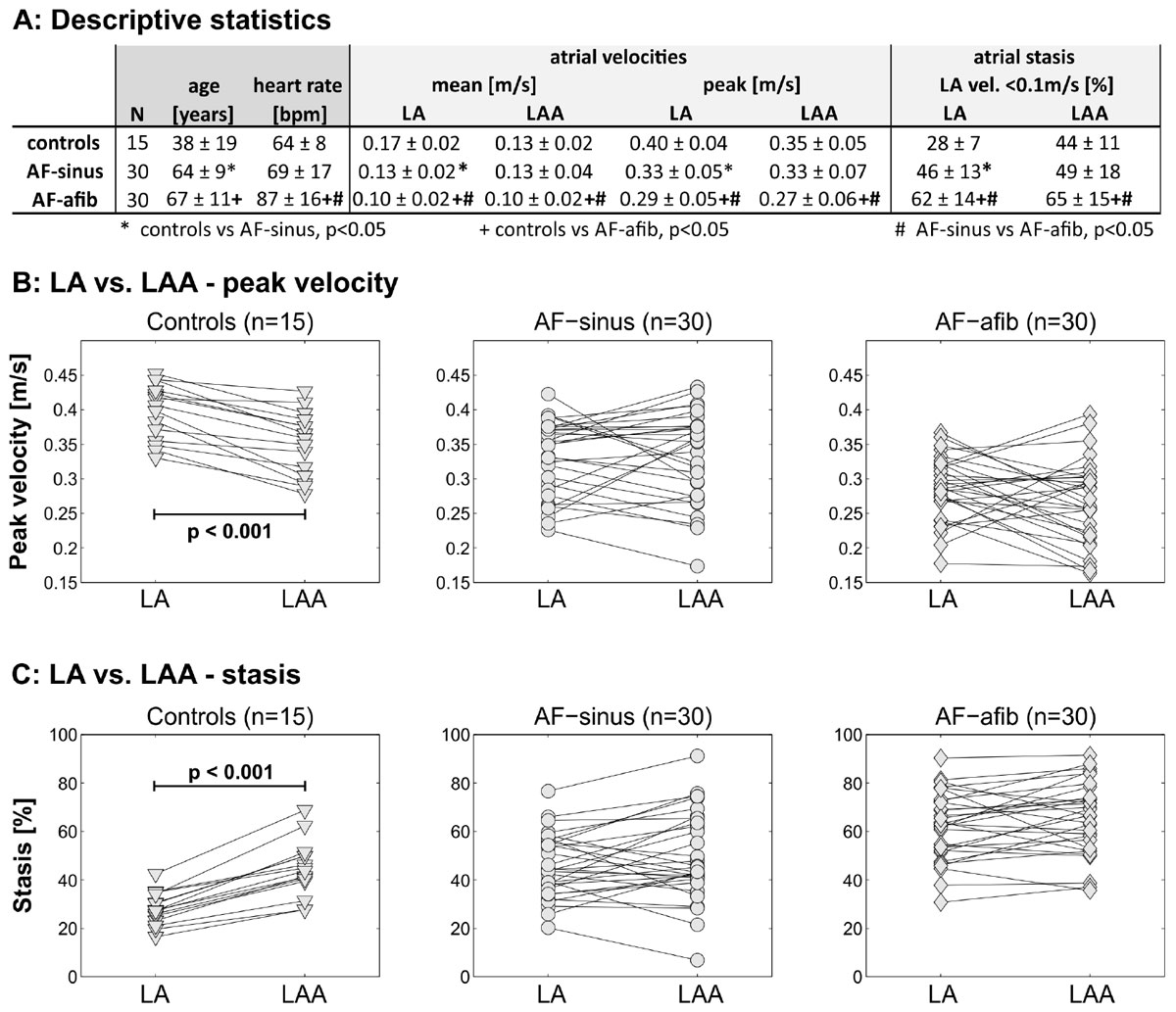
**A: Patient demographics and summary of LA and LAA flow quantification results**. B,C: Peak velocity and stasis in the LA compared to the LAA in n = 75 subjects (15 controls and 60 AF patients).

LA and LAA mean velocity, stasis, and peak velocity were significantly different between controls, AF-afib patients, and AF-sinus patients (all p < 0.001, figure 2A). TTP did not show consistent differences among groups. Comparison of LAA vs. LA flow dynamics revealed consistently decreased LAA mean and peak velocities by 21% and 12% (p < 0.0001) and increased LAA stasis by 58% (p < 0.0001) compared to the LA for all control subjects. In AF patients, differences between LAA and LA flow dynamics were less pronounced and did not show a clear trend (figure 2B and 2C). Notably, for all groups (controls and AF patients), LAA and LA flow dynamics were strongly correlated (LAA vs. LA mean velocity: r = 0.72; peak velocity: r = 0.64; stasis: r = 0.78, all p < 0.001).

## Conclusions

The findings of this study demonstrate that flow velocity and stasis measured in the LAA are strongly associated with the same measures in the LA. The absence of systematic differences in LAA vs. LA hemodynamics in AF patients suggests that presence of AF results in impaired flow dynamics (reduced velocities, increased stasis) not just in the LAA but in the in the entire left atrium. An individual assessment of LAA and LA flow may thus help to better identify patients at risk for thromboembolism beyond current clinical risk scores.

